# Is It Time to Review Guidelines for ETT Positioning in the NICU? SCEPTIC—Survey of Challenges Encountered in Placement of Endotracheal Tubes in Canadian NICUs

**DOI:** 10.1155/2016/7283179

**Published:** 2016-01-19

**Authors:** Pankaj Sakhuja, Michael Finelli, Judy Hawes, Hilary Whyte

**Affiliations:** ^1^Division of Neonatology, The Hospital for Sick Children, University of Toronto, Toronto, ON, Canada M5G 1X8; ^2^Department of Pediatrics, University of Toronto, Toronto, ON, Canada M5G 1X8; ^3^King Hamad University Hospital, Block 228, Busaiteen, Bahrain

## Abstract

*Objectives*. To examine current opinions and practices regarding endotracheal tube placement across several Canadian Neonatal Intensive Care Units.* Design*. Clinical directors from Canadian Neonatal Network affiliated NICUs and Neonatal-Perinatal Programs across Canada were invited via email to participate in and disseminate the online survey to staff neonatologists, neonatal fellows, respiratory therapists, and nurse practitioners.* Result*. There is wide variability in the beliefs and practices related to ETT placement. The majority use “weight +6” formula and “aim to black line” on ETT at vocal cords to estimate the depth of an oral ETT and reported estimation as challenging in ELBW infants. The majority agreed that mid-trachea is an ideal ETT tip position; however their preferred position on chest X-ray varied. Many believe that ETT positioning could be improved with more precise ETT markings.* Conclusion*. Further research should focus on developing more effective guidelines for ETT tip placement in the ELBW infants.

## 1. Introduction

Endotracheal intubation is a common procedure in the NICU and accurate positioning of the endotracheal tube (ETT) is essential to prevent associated morbidity. This is applied more so in cases of extremely premature babies where the trachea is much shorter, leaving little margin for error. Given that the difference between extubations and bronchial intubation can be less than a couple of centimeters, it is not surprising that the staff performing an emergency intubation are likely to insert the ETT deeper than required in order to avoid the risk of inadvertent extubations, especially during ETT taping; however this does give rise to an increased risk of right main stem intubation.

Many formulae have been proposed in an attempt to accurately estimate the depth of insertion of the ETT in order to place it at the mid-tracheal position. These are not always accurate, in particular at the extremes of low gestational age (ELGA) or in SGA infants when using body weight derived formulae. Compounding factors such as activity level, securing mechanisms, and route of intubation may impact the likelihood of malposition.

One study suggested gestational age based guidelines for ETT depth estimation and confirmed a significant reduction in the need for repositioning and incidence of uneven lung expansion [1, 2]. This has now been incorporated by the European, New Zealand, Australian, and UK resuscitation councils as standard [3–5].

We conducted this survey to examine the beliefs and current practices across Canada in regard to endotracheal tube placement and to understand the challenges faced by current practices. We hypothesized that there would be wide variability in practices in how to determine ETT placements across Canada. The majority would be using the weight based guidelines for ETT depth estimation and finding the estimation of depth of insertion challenging especially in infants with a birth weight <750 g. Medical practitioners are likely to insert ETT deeper than T2 to avoid the risk of extubations. Most respondents would also prefer frequent measurement markings on the ETT as a guide to correct placement. The results of the survey developed should act as a needs assessment and serve as the basis for future work in addressing issues related to ETT placement in neonates especially in the ELGA infant.

## 2. Methods

A cross-sectional survey of a sample of health care professionals involved in neonatal intubations across Canada was performed. Research Ethics Board approval was obtained from the Hospital for Sick Children. Clinical directors in the Canadian Neonatal Network affiliated NICUs and Neonatal-Perinatal Program Directors were invited to participate in and disseminate the survey invitation to their staff including neonatologists, neonatal fellows, registered respiratory therapists (RRTs), and nurse practitioners (NPs). Consent was implied by participation. Email reminders were sent at three and five weeks after initial email invitation.

The questionnaire was web based with 35 close ended questions requiring approximately 15 minutes to answer. It was devised using a modified Delphi process with input from a variety of health care professionals representing the participating disciplines. A pilot survey was administered to representatives of these stakeholder groups and modifications to the questionnaire were made based on this feedback. The questions are placed under 6 headings: personal experience, unit experience, challenges in depth estimation, positioning of ETT, challenges in positioning, and complications of malposition as shown in the Appendix. The results were analyzed using simple descriptive statistics.

## 3. Results

The clinical directors were identified from Canadian Neonatal Network affiliated NICUs and Neonatal-Perinatal Programs across Canada. They were invited via email to participate in and disseminate the survey. A total of 207 responses were received of which 85.5% were completed.

The representation of the various professions within the respondents is outlined in Table 1, with highest number being from RRTs. Clinical experience ranged from <5 to >15 years. Most respondents (48%) performed between 5 and 14 intubations per year. Majority of respondents (86%) worked in Level 3 NICUs and (76%) in combined inborn and out born NICUs. Intubations were performed almost equally among different respondent groups except staff which were involved in only 46% of the intubations as reported.

### 3.1. Estimation of ETT Depth and Ideal ETT Position

The majority of respondents (87%) used “weight +6” formula and “aim to black line” to estimate the depth of insertion of an oral ETT. Most respondents reported that they found the estimation of depth of insertion challenging in ELBW infants. Very few (22%) believed that the gestation age based guidelines may give better estimation of the depth of ETT insertion. Although the respondents (92%) identified mid-trachea as the ideal ETT tip position (Table 2), their preferred position on chest X-ray (CXR) varied considerably amongst them (Table 3). There was a wide variability of the reported practices (Table 4).

### 3.2. Complications of Malpositioning

Respondents felt that the most commonly seen complications of malpositioning were atelectasis (82%), differential air expansion (68%), unequal surfactant administration (61%), pneumothorax (29%), and PIE (14%).

Half of the respondents felt that more precise markings on the ETT would assist in better positioning of the ETT tip, although another 27% were unsure of the value. Most preferred markings every 5 mm.

## 4. Discussion

We found significant variability in the ETT placement practices across Canada. A few of the practices utilized could be further enhanced by having markings on ETT every 5 mm.

Endotracheal intubation is often performed in an emergent situation utilizing the weight based guide (weight +6) to estimate the insertion length of the ETT in order to be positioned mid trachea. Weight is often not available at the time of intubation at birth and a rough estimate is used to determine the insertion length. This is supplemented with the aim to position the black line of the ETT at the level of the vocal cords and produce bilateral equal air entry to guide its optimal placement. This has also been the recommendations of the American Academy of Pediatrics Neonatal Resuscitation Program without addressing the babies with birth weight less than 1000 gms [6].

Accurate positioning of the endotracheal tube (ETT) is essential to prevent associated morbidities, more so in cases of extreme premature babies where the trachea is much shorter, which leaves the clinicians with little margin for error. Therefore, satisfactory positioning of the tip of ET tube on initial intubation is extremely important. Weight is not a good predictor of the upper airway distances as shown in a postmortem study on 24 infants ranging from 23-week gestation to term plus 8 weeks [7]. It has also been shown that aiming to black line may not be the appropriate method to guide the placement of the ETT at mid-trachea, since it may place ETT too low in some or too high in others [8]. Auscultation of the bilateral breath sounds by itself does not rule out endobronchial intubation in children [9]. Even if the weight +6 (rule of 7-8-9) is supplemented with aim to black line and auscultation of the bilateral breath sounds optimal ETT placements in neonates remain a great challenge.

Within the neonatal literature, there is limited data regarding the ideal placement of the ETT tip within the trachea. Many have defined this level as the 1st or 2nd thoracic vertebrae [1, 2, 7, 10, 11].

The newborn larynx is positioned higher in the neck, it extends from C3 to C5 and carina is situated between T3 and T5 and most commonly at T4 [10, 12, 13]. If this information is to be collated, then the midpoint of the airway would be somewhere between T1 and T2. Wong et al. in 2008 published that the sternal notch to the carina represents 60% of the vocal cord to the carina distance [14]. T1 is located just below the sternal notch; it can thus be assumed that T1-T2 is the midpoint of the airway (vocal cord to the carina).

Tracheal position is affected by both respiratory movements and changes in head position such that flexion of neck and expiration shortens the distance pushing the ETT tip towards the carina and extension of the neck and inspiration retracts the ETT tip away from the carina [7]. Surprisingly we saw varied responses to the questions related to this. When extreme flexion or extension of the neck is expected after ETT insertion, the resultant change in the final position of ETT must be anticipated when deciding on the depth of ETT insertion. Rotschild et al. in 1991 suggested that the mid-tracheal position (midpoint of vocal cord to carina distance) is safe for both <1000 gm and >1000 gms infants based on 90th centile of changes in ETT position with maximum flexion and extension [7].

As the difference between extubations and bronchial intubation is only a matter of a couple of centimeters, more so in smaller babies, it is not surprising that the resident staff performing an emergency intubation are likely to insert the ETT deeper than required into the airway to avoid the risk of extubations with enhanced risk of right main stem intubation and/or atelectasis of right upper lobe of lung not to mention unilateral surfactant administration.

There has been no evidence to date that keeping the ETT tip between T1 and T2 is associated with the increase incidence of the unplanned extubations. On the contrary, T1-T2 has been reported as an ideal position for ETT tip [1, 2, 7, 10, 11].

The rule of 7-8-9 (weight +6) is universally followed to estimate the depth of an oral ETT to accurately place it at the mid-tracheal level. This rule was suggested by Tochen in 1979 who studied 40 neonates ranging from 26 to 44 weeks with the weight of 700–4100 grams and reference to T1-T2 as a mid-tracheal position. There were only ten babies under 1000 gms and none below 750 gms. They showed a linear relationship between tube length and weight with a correlation coefficient of 0.96 and assumed this even in babies <1000 gms [11]. Peterson et al. noticed that this rule predicted the tube length to be too long in infants below 750 gms but worked well for babies above this weight. Their study had just 5 infants between 750 and 1000 gms and just 16 infants above 3000 gms. They also used an incorrect landmark, with a point halfway between the inferior clavicle and carina on a chest radiograph as a mid-tracheal rather than T1-T2 [15]. Therefore, this rule may not apply in VLBW infants and in infants >2.5 kg. In babies with a weight <1000 grams, this rule overestimates the tube depth and may lead to complications related to malposition [1, 15]. In a published report the incidence of the ETT reposition after the initial placement in 23–26 weeks of gestation was 75% and of these 53% had uneven lung expansion. Weight based guidelines would result in the ETT length of 6.5–7 cms for infants of 500–1000 gms and this would include many infants of 23–27 weeks of gestation. This study suggested gestational age based guidelines for ETT depth estimation [2]. The same group validated these guidelines in a prospective audit and confirmed a significant reduction in the need for repositioning and incidence of uneven lung expansion [1]. ETT tip positioning although likely a minor but important contributor is worth consideration for the prevention of lung injury or bronchopulmonary dysplasia (BPD).

These gestational age based guidelines [2] have now been incorporated in the UK, Australian, and New Zealand Resuscitation Council recommendations [3, 4]. The American Academy of Pediatrics Neonatal Resuscitation Program recommends using vocal cord guide (aim to black line on ETT) to place the ETT approximately halfway between the vocal cords and the carina [6]. None of these guidelines specifically refers to T1-T2 as the ideal mid-tracheal position.

Most of the respondents also acknowledged that more precise marking on the ETT may help in better positioning of the ETT tube and prefer at least 5 mm markings. We strongly support this idea and feel this should be considered by the manufacturing companies.

## 5. Limitations

There are several limitations of this survey. The clinical directors and the Neonatal-Perinatal Program directors were approached to participate in and disseminate the survey to the specified groups working in their institute. We are unable to report the response rate as the exact number to whom the survey was sent out could not be ascertained and so the results may not represent all of the practices across Canada. However beliefs can form the basis of actions and the survey does highlight the issues related to challenges encountered in the ETT placement especially in the very preterm infant.

In conclusion, we noticed a wide variability in the beliefs related to ideal ETT placement across Canada. Our survey suggests that there is a real need for more research and consensus statement on the ideal position of the ETT with recognition that even a minor length difference may make a huge impact on the respiratory morbidity. Precise attention to ETT securing methods and need for regular review including chest X-ray should also be considered part of regular monitoring to ensure correct positioning once placement is verified. Industry should consider more frequent markings on the ETT to aid in better placement.

## Figures and Tables

**Table 1:** Representation of respondent.

Response	Percentage
Respiratory therapist	47.2%
Staff neonatologist	18.0%
Neonatology fellow	21.3%
Nurse practitioner	13.5%

**Table 2:** Ideal position of the ETT.

Response	Percentage
Upper trachea	3.9%
Mid-trachea	91.1%
Lower trachea	5.0%

**Table 3:** Ideal position on X-ray.

Response	Percentage
C7-T1	4.5%
T1-T2	34.7%
T2-T3	51.7%
T3-T4	5.1%
Unsure	4%

**Table 4:** Preferred practices.

Oral intubation	Unit (45%) transport (59%)

Premedication for elective/planned intubation	Always or almost always (combined 93%)

Methods to secure ETTs	(i) Tapes only (40%) (ii) Tape plus adhesive (i.e., tapes used with addition of adhesive like Mastisol to increase the adhesive strength) (38%) (iii) Sutures with tapes (11%) (iv) Adhesives with tapes and sutures (2%)

Other methods used for securing ETT	NeoBar, tapes with NeoBar, and NeoBridge

Point of measurement for an oral ETT	Upper lip (70%)

Confirming the ETT position	(i) 69% use 1 view (AP view) (ii) 77% also rely on auscultation of the breath sounds (iii) 19% also used other methods like end tidal co2 detectors, mist in the tube, chest rise, and clinical improvement

Reintubations (length same as before)	94% would not get an X-ray

Position of the head during the CXR	Neutral or midline (62%)

Analgesia/sedation during mechanical ventilation	Sometimes (66%)

Accidental extubations were reported	Occasionally by 76%

Knowledge about the level of the vocal cords and carina	Marked differences

Effects of flexion and extension on the ETT position	Marked differences

Auscultation of the bilateral breath sounds was not believed to rule out endobronchial intubations	70% agreed

Tube repositioning	(i) 81% felt the need to reposition the ETT sometimes (ii) T1-T2 26% will reposition (iii) T2-T3 7% did not reposition

## Appendix

*(1) Personal Experience*

*Question 1*

What is your occupation?
  ◯ Respiratory Therapist
  ◯ Staff Neonatologist
  ◯ Neonatology Fellow
  ◯ Nurse Practitioner

*Question 2*

How many years of NICU experience do you have?
  ◯ <5
  ◯ 5–9 years
  ◯ 10–14 years
  ◯ >15 years

*Question 3*

On average how many intubations do you perform per year?
  ◯ <5
  ◯ 5–14
  ◯ 15–25
  ◯ >25

* (2) Unit Experience*

*Question 4*

What is the Level of your NICU?
  □ Level 1
  □ Level 2
  □ level 2 (advanced)
  □ Level 3

*Question 5*

Type of NICU?
  □ Inborn
  □ Outborn
  □ Both

*Question 6*

Who routinely performs intubations in the unit? (tick all that apply)
  □ Resident
  □ Fellows
  □ Staff (consultant)
  □ Respiratory therapist
  □ Nurse Practitioners
  □ Other (specify) ______________________

*Question 7*

What types of intubation are performed in your unit?
  □ Predominantly oral
  □ Predominantly nasal
  □ Both

*Question 8*

What type of intubations do you perform for neonatal transport?
  □ Primarily oral
  □ Primarily nasal

*Question 9*

Do you premedicate for elective/planned intubations (e.g., opioid and/or muscle relaxation)?
  □ Always
  □ Almost always
  □ Sometimes
  □ Almost never
  □ Never

*Question 10*

How often do you use analgesia and/or sedation during mechanical ventilation?
  □ Always
  □ Almost always
  □ Sometimes
  □ Almost never
  □ Never

*Question 11*

How do you secure your ETTs?
  □ Tapes only
  □ Tape plus liquid adhesive (i.e., Mastisol).
  □ Tapes plus suture through ETT.
  □ Tapes, liquid adhesive (i.e., Mastisol) plus suture through ETT.
  □ Other – please specify ______________________

*Question 12*

In my unit, unintentional extubations are seen?
  □ Often
  □ Occasionally
  □ Almost never
  □ Never

* (3) Challenges in Depth Estimation*

*Question 13*

How do you estimate the depth of insertion of an oral ETT? (tick all that apply)
  □ Weight +6 cm
  □ Gestational age estimate
  □ Other weight estimate
  □ Aim to black line
  □ Other (specify) ______________________
  □ Not applicable

*Question 14*

How do you estimate depth of insertion for a nasal ETT? (tick all that apply)
  □ Weight +7 cm
  □ Gestational age estimate
  □ Other weight estimate
  □ Aim to black line
  □ Other (specify) ______________________
  □ Not applicable

*Question 15*

I find estimating the depth of ETT insertion challenging in (tick all that apply)
  □ Infants
  □ 750–999 g
  □ 1–1999 kg
  □ 2-3 kg
  □ >3 kg
  □ None of the above
  □ Unsure

*Question 16*

Do you think narrower gestational age calculations may give better estimation of the depth of ETT tube?
  □ Yes
  □ No
  □ Unsure

*Question 17*

More precise weight adjusted calculations for ETT depth estimation may be useful in (tick all that apply)
  □ Infants
  □ 750–999 g
  □ 1–1999 g
  □ 2-3 kg
  □ >3 kg
  □ None of above
  □ Unsure

* (4) Positioning of ETT*

*Question 18*

From what point do you calculate the oral ETT measurement?
□ Upper Gum
□ Upper Lip
□ Others ______________________
□ Not applicable

*Question 19*

Which do you think is a better point for measurement of an oral ETT?
□ Upper Gum
□ Upper Lip
□ Others ______________________
□ Not applicable

*Question 20*

What in your opinion is the ideal position of the ETT tip in a neonate?
□ Upper Trachea
□ Mid Trachea
□ Lower Trachea

*Question 21*

The ideal position of the ETT tip on X-Ray is
  □ C7-T1
  □ T1-T2
  □ T2-T3
  □ T3-T4
  □ Unsure

*Question 22*

The Level of vocal cords in neonates is
  □ C3-C4
  □ C4-C5
  □ C5-C6
  □ C6-C7
  □ C7-T1
  □ Unsure

*Question 23*

The level of carina in a neonate is. (Tick all that applies)
  □ T3-T4
  □ T4-T5
  □ T3–T5 (mostly at T4)
  □ T5-T6
  □ T4–T6 (mostly at T5)
  □ Unsure

*Question 24*

Flexion of the neck
  □ Pushes the ETT down
  □ Pulls the ETT up
  □ Does not impact
  □ Unsure

*Question 25*

Extension of the neck
  □ Pushes the ETT down
  □ Pulls the ETT up
  □ Does not impact
  □ Unsure

*Question 26*

How do you confirm ETT position? (Tick all that applies)
  □ Chest X-ray – 2 views (AP & Lateral)
  □ Chest X-ray – 1 view (AP only)
  □ Chest X-ray 1 view (Lateral only)
  □ Ultrasound
  □ Auscultation of breath sounds
  □ Other (specify) ______________________

*Question 27*

How often do you perform a CXR after intubation?
  □ Always
  □ Almost always
  □ Almost never
  □ Never

*Question 28*

I do not perform a CXR post intubation if (tick all that apply)
  □ Air entry sounds equal
  □ Improvement in clinical status
  □ Colour change with Co2 detector
  □ Measurement appropriate by weight +6/7 rule
  □ Measurement appropriate by gestational age estimates
  □ Previously intubated and ETT measurement same

*Question 29*

Do you have specific head positional requirements for the infant during CXR
  □ Yes
  □ No
  □ If yes, please specify ______________________

*Question 30*

In my opinion auscultation of bilateral equal breath sounds rules out right mainbronchus intubation?
□ Yes
□ No
□ Unsure

* (5) Challenges in Positioning*

*Question 31*

How often do you have to reposition your ETT after X-ray?
□ Always
□ Almost always
□ Sometimes
□ Almost never
□ Never
□ Unsure

*Question 32*

I would reposition the ETT if on the chest X-Ray it is at (tick all that apply)
  □ C7-T1
  □ T1-T2
  □ T2-T3
  □ T3-T4
  □ T5 and below

*Question 33*

Do you think better positioning of the ETT tip could be achieved with more precise markings on ETT (current markings every 10 mm)
  □ Yes
  □ No
  □ Unsure

*Question 34*

If yes, what markings would you prefer to see?
  □ Every 2 mm
  □ Every 5 mm
  □ Others, Please specify ______________________
  □ Not applicable

* (6) Complication of Malpositioning*

*Question 35*

Complications of ETT malpositioning I most commonly see are (tick all that apply)
  □ Unequal surfactant administration
  □ Differential Air expansion
  □ Atelectasis
  □ Pneumothorax
  □ PIE
  □ Others ______________________
